# Supplementary material for: Electron energy loss spectroscopy database synthesis and automation of core-loss edge recognition by deep-learning neural networks
Source: Sci Rep. 2022 Dec 23;12:22183. doi: 10.1038/s41598-022-25870-3 (PMC9789080; doi:10.1038/s41598-022-25870-3)
Supplement: Supplementary file 2 — Supplementary Table S2. [file 41598_2022_25870_MOESM2_ESM.pdf]

Table S2 Experimental Conditions of Real Spectra Dataset

ID	Spectrum Name	Edge Type	Material Analyzed	Microscope Name	Gun Type	Incident Bea	Reolution (eV)	Monochroma	Dispersion (eV/pix	Acquisition Mode	Collection Semi-an	Integration Time (se	Detector	Calibration	Relative Thickness (t/λ)
1	Polyethylene (HDPE)	C-K	(C2H4)n	JEOL JEM 2100F	FEG	120 kV	0.9 eV	No	0.05 eV/pixel	Imaging	100 mrad	10 secs	Gatan Enfina	zero+dispersion	0.59 t/λ
2	Polylactic Acid	C-K	(C3H4O2)n	JEOL JEM 2100F	FEG	120 kV	0.9 eV	No	0.05 eV/pixel	Imaging	100 mrad	10 secs	Gatan Enfina	zero+dispersion	0.74 t/λ
3	Polypropylene	C-K	(C3H6)n	JEOL JEM 2100F	FEG	120 kV	0.9 eV	No	0.05 eV/pixel	Imaging	100 mrad	10 secs	Gatan Enfina	zero+dispersion	0.54 t/λ
4	LSMO Perovskite	Mn-L23	(La0.7,Sr0.3)MnO3	STEM-VG	Cold FEG	100 kV			0.229 eV/pixel	STEM	22 mrad	0.2 secs	Parallel: 666 + CCD modified Orsay		
5	LSMO Perovskite	O-K	(La0.7,Sr0.3)MnO4	STEM-VG	Cold FEG	100 kV			0.213 eV/pixel	STEM	23 mrad	0.2 secs	Parallel: 666 + CCD modified Orsay		
6	Amorphous Carbon 500ºC, 15 Min	C-K	a-C:H	Tecnai F30	Schottky	300 kV	1.2 eV	No	0.05 eV/pixel	STEM	5.9 mrad		Gatan Tridiem 863	Pi* at 285 eV	
7	Oxidised Amorphous Carbon, 500ºC, 2500 Min	C-K	a-C:H,O	Tecnai F30	Schottky	300 kV	1.2 eV	No	0.05 eV/pixel	STEM	5.9 mrad		Gatan Tridiem 863	Pi* at 285 eV	
8	Silicon Dioxide Amorphous	Si-L23	a-SiO2	CM20	LaB6	100 kV	1.08 eV		0.197 eV/pixel	Diffraction	6.14 mrad		Parallel: 666 Gatan		0.5 t/λ
9	Silicon Dioxide Amorphous	Si-L23	a-SiO3	CM20	LaB6	100 kV	1.08 eV		0.099 eV/pixel	Diffraction	6.14 mrad	1.6 secs	Parallel: 666 Gatan		0.5 t/λ
10	Aluminium Nitride	N-K	AlN	CM30	LaB6	100 kV	1.6 eV		0.1 eV/pixel	Diffraction	5 mrad	1.504 secs	Parallel		0.5 t/λ
11	Iron (III) Oxide, Hematite	Fe-L23	alpha-Fe2O3	JEOL JEM-2100F-UHR	feg Schottky	200 kV			0.1 eV/pixel	Imaging	16 mrad	20 secs	GIF: GIF 2001		
12	Pure Boron	B-K	B	STEM-VG	Cold FEG	100 kV	0.7 eV		0.477 eV/pixel	STEM	24 mrad		Parallel: 666 Gatan		
13	Boron Carbide	B-K	B4C	CM30	LaB6	100 kV	1.2 eV	No	0.1 eV/pixel		5 mrad	1 secs	Parallel: 666 Gatan		0.6 t/λ
14	Boron Carbide	C-K	B4C	CM30	LaB6	100 kV	1.2 eV	No	0.1 eV/pixel		5 mrad	1 secs	Parallel: 666 Gatan		0.6 t/λ
15	Boron Nitride Single Wall Nanotube	B-K, N-K	BN	STEM-VG	Cold FEG	100 kV	0.7 eV	No	0.477 eV/pixel		24 mrad		Parallel: 666 Gatan		
16	Boron Nitride Multiwall Nanotube	N-K	BN	STEM-VG	Cold FEG	100 kV	0.7 eV		0.477 eV/pixel	STEM	24 mrad		Parallel: 666 Gatan		
17	Boron Nitride Multiwall Nanotube	N-K	BN	STEM-VG	Cold FEG	100 kV	0.7 eV		0.477 eV/pixel	STEM	24 mrad		Parallel: 666 Gatan		
18	Hexagonal Boron Nitride	B-K, N-K	BN(hex)	JEOL 3000F	feg Schottky	300 kV	1.2 eV	No	0.5 eV/pixel		2.55 mrad	0.2 secs	Parallel: GIF2000		0.788 t/λ
19	Diamond	C-K	C	CM20	LaB6	100 kV	1.8 eV		0.1 eV/pixel	Diffraction	5 mrad	0.4 secs	Parallel: 666 Gatan		0.6 t/λ
20	Graphite	C-K	C	CM30	LaB6	100 kV	1.2 eV		0.1 eV/pixel	Diffraction	5 mrad	0.5 secs	Parallel: 666 Gatan		0.37 t/λ
21	Carbon Single Wall Nanotube	C-K	C	STEM-VG	Cold FEG	80 kV	0.5 eV		0.2 eV/pixel	STEM	6 mrad		Parallel: 666 Gatan		
22	Reduced Graphene Oxide	C-K	C	Tecnai F30	feg Schottky	300 kV		No	0.3 eV/pixel		136 mrad		GIF: GIF		
23	Graphite (really Glassy Carbon)	C-K	C (graphite)	VG HB501A	Cold FEG	100 kV	0.5 eV		0.088 eV/pixel	STEM	16 mrad	10 secs	GIF:		
24	Cubic Boron Nitride	B-K	c-BN	Jeol 4000FX	LaB6	400 kV	1.8 eV		0.1 eV/pixel	Diffraction	1.5 mrad		Parallel: 666 Gatan		0.275 t/λ
25	Cubic Boron Nitride	N-K	c-BN	Jeol 4000FX	LaB6	400 kV	1.8 eV		0.1 eV/pixel	Diffraction	1.5 mrad		Parallel: 666 Gatan		0.275 t/λ
26	Oxidized-graphenic Nanoplatelets Obtained From Bamboo – High Oxygen	C-K	C-O	FEI Titan Cube	FEG	80 kV	0.6 eV	No	0.05 eV/pixel		20 mrad		Gatan Tridiem	Pi* at 285 eV	
27	Oxidized-graphenic Nanoplatelets Obtained From Bamboo – High Oxygen	C-K, O-K	C-O	FEI Titan Cube	FEG	80 kV	0.8 eV	No	0.201 eV/pixel		20 mrad		Gatan Tridiem	Pi* at 285 eV	
28	Melamine Powder	C-K, N-K	C3N6H6	VG HB501	Cold FEG	100 kV		No	0.206 eV/pixel		24 mrad	0.090 secs	Parallel: 666 Gatan		
29	Melamine Powder -Irradiated	C-K, N-K	C3N6H6	VG HB501	Cold FEG	100 kV		No	0.208 eV/pixel		24 mrad		Parallel: 666 Gatan		
30	CaCoO Misfit Nanotube	Co-L23, O-K, Ca-L23	CaCoO2−CoO2	FEI Titan Low-Base	X-FEG	200 kV	1.5 eV	No	0.526 eV/pixel	STEM	10 mrad		Gatan Tridiem ESR 865	low loss + drift tube	
31	Calcium Titanate (perovskite Structure)	Ca-L23	CaTiO3	JEOL2010F	feg Schottky	200 kV	1 eV		0.2 eV/pixel	Diffraction	5 mrad	5 secs	Parallel: 766 Gatan		0.3 t/λ
32	Calcium Titanate (perovskite Structure)	Ti-L23, O-K	CaTiO3	JEOL2010F	feg Schottky	200 kV	1 eV	No	0.2 eV/pixel		5 mrad	5 secs	Parallel: 766 Gatan		0.3 t/λ
33	CN Crystal	C-K, N-K	CN	VG HB501	Cold FEG	100 kV	0.7 eV	No	0.203 eV/pixel		24 mrad	0.2 secs	Parallel: 666 Gatan		
34	Cobalt	Co-L23	Co	STEM-VG	cold field emission	100 kV	0.6 eV		0.202 eV/pixel	STEM	22 mrad	0.3 secs	Parallel: 666 + CCD modified Orsay		
35	Metallic Cobalt	Co-L23	Co	JEOL2010F	feg Schottky	200 kV		No	0.5 eV/pixel		28 mrad		GIF: GIF		
36	Cobalt (III) Oxide (IV)	Co-L23	Co3O4	Tecnai F20 monochrom	feg	200 kV	0.35 eV	Yes	0.05 eV/pixel		3 mrad	25 secs	Parallel: GIF Tridiem		
37	Cobalt(II,III) Oxide	Co-L23	Co2O3	JEOL2010F	feg Schottky	200 kV		No	0.5 eV/pixel		28 mrad	5 secs	GIF: GIF		
38	Cobalt Oxide	Co-L23	CoO	Tecnai F20 monochrom	feg	200 kV	0.3 eV	Yes	0.05 eV/pixel			15 secs	Parallel: GIF Tridiem		
39	Cobalt Oxide	O-K	CoO	STEM-VG	cold field emission	100 kV	0.6 eV		0.206 eV/pixel	STEM	22 mrad	0.3 secs	Parallel: 666 + CCD modified Orsay		
40	Cobalt Oxide	Co-L23	CoO	STEM-VG	cold field emission	100 kV	0.6 eV		0.206 eV/pixel	STEM	22 mrad	0.3 secs	Parallel: 666 + CCD modified Orsay		
41	Cobalt Monoxide	Co-L23	CoO	JEOL2010F	feg Schottky	200 kV		No	0.5 eV/pixel		28 mrad	5 secs	GIF: GIF		
42	Chromium Oxide (Chromia)	O-K, Cr-L23	Cr2O3 (alpha)	Philips CM200	feg Schottky	197 kV	0.8 eV	No	0.1 eV/pixel		5.9 mrad		GIF: GIF200		0.2 t/λ
43	Chromium Carbide	Cr-L23	Cr3C2	HB501	feg	100 kV			0.1 eV/pixel	STEM	20 mrad	4 secs	Parallel: 666 Gatan		
44	Chromium Carbide	C-K	Cr3C2	HB501	feg	100 kV			0.1 eV/pixel	STEM	20 mrad	4 secs	Parallel: 666 Gatan		
45	Chromium Carbide	Cr-L23	Cr7C3	HB501	FEG	100 kV			0.1 eV/pixel	STEM		8 secs	Parallel: 666 Gatan		
46	Chromium Carbide	C-K	Cr7C3	HB502	FEG	100 kV			0.1 eV/pixel	STEM		8 secs	Parallel: 666 Gatan		
47	Chromium Monocarbide	C-K, O-K, Cr-L2,3	CrC	VG HB501UX	Cold FEG	100 kV	0.8 eV	No	0.3 eV/pixel		6.5 mrad	5 secs	Parallel: Gatan ENFINA		
48	Copper	Cu-L2,3	Cu	CM20	LaB6	100 kV			0.1 eV/pixel	Diffraction	3 mrad		Parallel: 666 Gatan		
49	Copper Oxide	Cu-L2,3	Cu2O	CM20	LaB6	100 kV			0.1 eV/pixel	Diffraction	3 mrad		Parallel: 666 Gatan		
50	Copper Oxide	O-K	Cu2O	CM20	LaB6	100 kV			0.1 eV/pixel	Diffraction	3 mrad		Parallel: 666 Gatan		
51	Cu L23 Edges In Cu4O3	Cu-L2,3	Cu4O3	ARM-200F	Cold	200 kV	0.45 eV	No	0.05 eV/pixel	Diffraction	18 mrad		GIF quantum ER	zero+dispersion in Dual EELS	0.6 t/λ
52	O-K Edge In Cu4O3	O-K	Cu4O3	ARM-200F	Cold	200 kV	0.45 eV	No	0.05 eV/pixel	Diffraction	18 mrad		GIF quantum ER	zero+dispersion in Dual EELS	0.6 t/λ
53	Copper Oxide	O-K	CuO	CM20	LaB6	100 kV			0.1 eV/pixel	Diffraction	3 mrad		Parallel: 666 Gatan		
54	Copper Oxide	Fe-L23	CuO	CM20	LaB6	100 kV			0.1 eV/pixel	Diffraction	3 mrad		Parallel: 666 Gatan		
55	Iron	Fe-L23	Fe	JEOL JEM-2100F-UHR	feg Schottky	200 kV			0.1 eV/pixel	Imaging	16 mrad	20 secs	GIF: GIF 2001		
56	Iron Oxide	Fe-L23	Fe2O3	Tecnai F20 monochrom	feg	200 kV	0.3 eV	Yes	0.05 eV/pixel			15 secs	Parallel: GIF Tridiem		
57	Hematite	Fe-L23	Fe2O3 (alpha)	Philips CM200	feg Schottky	197 kV	0.8 eV		0.1 eV/pixel	Diffraction	5.9 mrad	15 secs	GIF: GIF200		
58	Iron Oxide Hematite	Fe-L23	Fe2O3 (alpha)	STEM-VG	Cold FEG	100 kV		No	0.05 eV/pixel		24 mrad		Parallel: 666 Gatan		
59	Iron Oxide Hematite	O-K	Fe2O3 (alpha)	STEM-VG	Cold FEG	100 kV		No	0.05 eV/pixel		24 mrad		Parallel: 666 Gatan		
60	FeAl	Fe-L23	FeAl	ARM 200cF	Cold Field Emission Gun	200 kV	0.7 eV	No	0.25 eV/pixel		33.19 mrad		GIF Quantum ER		
61	Iron Oxide Siderite	Fe-L23	FeCO3	STEM-VG	Cold FEG	100 kV		No	0.05 eV/pixel		24 mrad		Parallel: 666 Gatan		
62	Iron Oxide Siderite	O-K	FeCO3	STEM-VG	Cold FEG	100 kV		No	0.05 eV/pixel		24 mrad		Parallel: 666 Gatan		
63	Iron Oxide 2-lines Ferrihydrite	Fe-L23	FeOOH	STEM-VG	Cold FEG	100 kV			0.05 eV/pixel		24 mrad		Parallel: 666 Gatan		
64	Iron Oxide 2-lines Ferrihydrite	O-K	FeOOH	STEM-VG	Cold FEG	100 kV			0.05 eV/pixel		24 mrad		Parallel: 666 Gatan		
65	Iron Oxide Goethite	Fe-L23	FeOOH (alpha)	STEM-VG	Cold FEG	100 kV		No	0.05 eV/pixel		24 mrad		Parallel: 666 Gatan		
66	Iron Oxide Goethite	O-K	FeOOH (alpha)	STEM-VG	Cold FEG	100 kV		No	0.05 eV/pixel		24 mrad		Parallel: 666 Gatan		
67	Iron Oxide Cl-containing Akaganeite	Fe-L23	FeOOH (beta)	STEM-VG	Cold FEG	100 kV		No	0.05 eV/pixel		24 mrad		Parallel: 666 Gatan		
68	Iron Oxide Cl-containing Akaganeite	O-K	FeOOH (beta)	STEM-VG	Cold FEG	100 kV		No	0.05 eV/pixel		24 mrad		Parallel: 666 Gatan		
69	Ferrous Titanate	Fe-L23	FeTiO3	Tecnai F20 monochrom	feg	200 kV	0.3 eV	Yes	0.05 eV/pixel			15 secs	Parallel: GIF Tridiem		
70	Iron Titanium Oxide	Fe-L23	FeTiO3	JEOL JEM-2100F-UHR	feg Schottky	200 kV			0.1 eV/pixel	Imaging	16 mrad	20 secs	GIF: GIF 2001		
71	La0.7Sr0.3MnO3/SrTiO3-(001) Heterostructure	Mn-L23	La0.7Sr0.3MnO3	FEI Titan 80-300ST	Field Emission Gun	300 kV	1.0 eV	No	0.2 eV/pixel		33.1 mrad		GIF	zero + dispersion	
72	La0.7Sr0.3MnO3/SrTiO3-(001) Heterostructure, Oxygen Vacancies	Mn-L23	La0.7Sr0.3MnO3	FEI Titan 80-300ST	Field Emission Gun	300 kV	1.0 eV	No	0.2 eV/pixel		33.1 mrad		GIF	zero + dispersion	
73	La0.7Sr0.3MnO3 Film On SrTiO3-(111) Substrate	Mn-L23, O-K	La0.7Sr0.3MnO3	ARM 200cF	Cold Field Emission Gun	200 kV	0.7 eV	No	0.25 eV/pixel	STEM	33.19 mrad		GIF Quantum ER	zero + dispersion	
74	La0.7Sr0.3MnO3/SrTiO3-(001) Heterostructure	O-K	La0.7Sr0.3MnO3	FEI Titan 80-300ST	Field Emission Gun	300 kV	1.0 eV	No	0.2 eV/pixel	STEM	33.1 mrad			PCA + background subtraction	
75	La0.7Sr0.3MnO3/SrTiO3-(001) Heterostructure, Oxygen Vacancies	O-K	La0.7Sr0.3MnO3	FEI Titan 80-300ST	Field Emission Gun	300 kV	1.0 eV	No	0.2 eV/pixel	STEM	33.1 mrad		GIF	PCA + background subtraction	
76	La0.7Sr0.3MnO3 Thin Film	Mn-L23	La0.7Sr0.3MnO3	ARM 200cF	Cold Field Emission Gun	80 kV	0.7 eV	No	2457.271 eV/pixel		66 mrad		GIF Quantum ER	Energy scaled aligned using zero loss peak. Power law background removed.	
77	La0.7Sr0.3MnO3 Thin Film, Electron Beam Damage	Mn-L23	La0.7Sr0.3MnO3	ARM 200cF	Cold Field Emission Gun	80 kV	0.7 eV	No	1982.691 eV/pixel		66 mrad		GIF Quantum ER	Energy scaled aligned using zero loss peak. Power law background removed.	
78	La0.7Sr0.3MnO3 Thin Film	O-K	La0.7Sr0.3MnO3	ARM 200cF	Cold Field Emission Gun	80 kV	0.7 eV	No	1525.632 eV/pixel		66 mrad		GIF Quantum ER	Energy scaled aligned using zero loss peak. Power law background removed.	
79	La0.7Sr0.3MnO3 Thin Film, Electron Beam Damage	O-K	La0.7Sr0.3MnO3	ARM 200cF	Cold Field Emission Gun	80 kV	0.7 eV	No	1347.815 eV/pixel	STEM	66 mrad		GIF Quantum ER	Energy scaled aligned using zero loss peak. Power law background removed.	
80	La0.7Sr0.3MnO3 Thin Film	Mn-L23	La0.7Sr0.3MnO3	ARM 200cF	Cold Field Emission Gun	200 kV	0.7 eV	No	649.245 eV/pixel		66 mrad		GIF Quantum ER	Energy scaled aligned using zero loss peak. Power law background removed.	
81	La0.7Sr0.3MnO3 Thin Film, Heavy Electron Beam Damage	Mn-L23	La0.7Sr0.3MnO3	ARM 200cF	Cold Field Emission Gun	200 kV	0.7 eV	No	610.026 eV/pixel		66 mrad		GIF Quantum ER	Energy scaled aligned using zero loss peak. Power law background removed.	
82	La0.7Sr0.3MnO3 Thin Film	O-K	La0.7Sr0.3MnO3	ARM 200cF	Cold Field Emission Gun	200 kV	0.7 eV	No	426.354 eV/pixel		66 mrad		GIF Quantum ER	Energy scaled aligned using zero loss peak. Power law background removed.	
83	La0.7Sr0.3MnO3 Thin Film, Electron Beam Damage	O-K	La0.7Sr0.3MnO3	ARM 200cF	Cold Field Emission Gun	80 kV	0.7 eV	No	352.864 eV/pixel	STEM	66 mrad		GIF Quantum ER	Energy scaled aligned using zero loss peak. Power law background removed.	
84	La2NiO4 – Structure Of K2NiF4	O-K, Ni-L23	La2NiO4	FEI TITAN THEMIS	XFEG	200 kV	0.25 eV	No	0.25 eV/pixel	STEM			Quantum GATAN Filter (STEM - Fas	low loss + drift tube	
85	O-K Edge – La2NiO4 – Structure Of K2NiF4 Tetragonale (I4/mmm)	O-K	La2NiO4	FEI TITAN THEMIS	XFEG	200 kV	0.1 eV	No	0.1 eV/pixel	STEM	33.87 mrad		Gatan Quantum Filter (FAST Dual EE	zero loss peak + dispersion	
86	Lanathanum Cobalt Oxide	O-K, Co-L23	LaCoO3	Philips CM20	LaB6	200 kV	2 eV	No	0.5 eV/pixel		7.5 mrad	20 secs	Parallel: GIF200		1 t/λ
87	LaFeO3 Thin Film	Fe-L23	LaFeO3	ARM 200cF	Cold Field Emission Gun	80 kV	0.7 eV	No	2403.014 eV/pixel		66 mrad		GIF Quantum ER	Energy scaled aligned using zero loss peak. Power law background removed.	
88	LaFeO3 Thin Film	Fe-L23	LaFeO3	ARM 200cF	Cold Field Emission Gun	80 kV	0.7 eV	No	0.25 eV/pixel		66 mrad		GIF Quantum ER		
89	Magnesium Carbonate	O-K	MgCO3	Philips CM20	LaB6	200 kV	1.2 eV	No	0.1 eV/pixel		7.6 mrad		Parallel: GIF		
90	Magnesium Carbonate	C-K	MgCO3	Philips CM20	LaB6	200 kV	1.2 eV	No	0.1 eV/pixel		7.6 mrad		Parallel: GIF		
91	Magnesium Oxide	O-K	MgO	CM30	feg Schottky	300 kV	1 eV		1 eV/pixel	Diffraction	5.21 mrad	0.4 secs	Parallel: GIF200		0.189 t/λ
92	Magnesium Oxide	O-K	MgO	STEM-VG	cold field emission	100 kV	0.6 eV	No	0.2 eV/pixel		22 mrad	0.2 secs	Parallel: 666 + CCD modified Orsay		
93	N2 Gas	N-K	N2	VG HB501	Cold FEG	100 kV		No	0.205 eV/pixel		24 mrad	0.6 secs	Parallel: 666 Gatan		
94	99.99% Nickel Metal	Ni-L23	Ni	CM30	feg Schottky	300 kV	1.1 eV		0.05 eV/pixel	Diffraction	3.5 mrad	10 secs	Parallel: GIF		0.5 t/λ
95	Nickel Monoxide	Ni-L23	NiO	CM30	feg Schottky	300 kV	1.1 eV		0.05 eV/pixel	Diffraction	3.5 mrad	10 secs	Parallel: GIF		0.5 t/λ
96	Silicon	Si-L23	Si	CM20	LaB6	100 kV	1.08 eV		0.197 eV/pixel	Diffraction	6.14 mrad		Parallel: 666 Gatan		0.4 t/λ
97	Silicon Nitride	Si-L23, N-K	Si3N4	VG HB501UX	Cold FEG	100 kV	1 eV	No	0.5 eV/pixel		6.5 mrad	0.8 secs	Parallel: Gatan ENFINA		0.46 t/λ
98	Silicon Nitride Alpha	Si-L23	Si3N4 (alpha)	CM30	LaB6	100 kV	1.1 eV		0.1 eV/pixel	Diffraction	10 mrad	0.18 secs	Parallel: 666 Gatan		0.4 t/λ
99	Silicon Nitride Alpha	Si-L23	Si3N4 (alpha)	CM30	LaB6	100 kV	1.1 eV		0.2 eV/pixel	Diffraction	10 mrad		Parallel: 666 Gatan		0.4 t/λ
100	Silicon Carbide 6H(hexagonal)	Si-L23	SiC(6H)	CM30	LaB6	100 kV	1 eV		0.1 eV/pixel	Diffraction	10 mrad	0.28 secs	Parallel: 666 Gatan		0.4 t/λ
101	Silicon Carbide 6H(hexagonal)	Si-L23	SiC(6H)	CM30	LaB6	100 kV	1 eV		0.2 eV/pixel	Diffraction	10 mrad		Parallel: 666 Gatan		0.4 t/λ
102	SrCoO Misfit NT	O-K, Co-L23	SrCoO	FEI Titan Low-Base	X-FEG	200 kV	2 eV	No	0.518 eV/pixel	STEM	17 mrad		Gatan Tridiem ESR 865		
103	Strontium Ferrite	Fe-L23	SrFeO3	JEOL JEM-2100F-UHR	feg Schottky	200 kV			0.1 eV/pixel	Imaging	16 mrad	20 secs	GIF: GIF 2001		
104	Strontium Titanate	Ti-L23	SrTiO3	JEOL 3000F	feg Schottky	300 kV	1.2 eV	No	0.3 eV/pixel		6.8 mrad	0.5 secs	Parallel: GIF2000		0.544 t/λ
105	Strontium Titanate	Ti-L23	SrTiO3	STEM-VG	Cold FEG	100 kV			0.212 eV/pixel	STEM	22 mrad	0.1 secs	Parallel: 666 + CCD modified Orsay		
106	Strontium Titanate	O-K	SrTiO3	STEM-VG	Cold FEG	100 kV			0.242 eV/pixel	STEM	22 mrad	0.3 secs	Parallel: 666 + CCD modified Orsay		
107	SrTiO3	Ti-L23, O-K	SrTiO3	ARM 200cF	Cold Field Emission Gun	200 kV	0.7 eV	No	0.25 eV/pixel	STEM	33.19 mrad		GIF Quantum ER	zero + dispersion	
108	SrTiO3-(001)	O-K	SrTiO3	FEI Titan 80-300ST	Field Emission Gun	300 kV	1.0 eV	No	0.2 eV/pixel	STEM	33.1 mrad		GIF	PCA + background subtraction	
109	SrTiO3-(111)	Ti-L23	SrTiO3	ARM 200cF	Cold Field Emission Gun	80 kV	0.7 eV	No	0.05 eV/pixel	STEM	66 mrad		GIF Quantum ER	zero + dispersion	
110	SrTiO3-(111)	Ti-L23	SrTiO3	ARM 200cF	Cold Field Emission Gun		0.7 eV	No	2619.52 eV/pixel		66 mrad		GIF Quantum ER	Energy scaled aligned using zero loss peak. Power law background removed.	
111	SrTiO3-(111), Light Beam Damage	Ti-L23	SrTiO3	ARM 200cF	Cold Field Emission Gun	200 kV	0.7 eV	No	1976.193 eV/pixel		66 mrad		GIF Quantum ER	Energy scaled aligned using zero loss peak. Power law background removed.	
112	SrTiO3-(111), Heavy Beam Damage	O-K	SrTiO3	ARM 200cF	Cold Field Emission Gun	200 kV	0.7 eV	No	375.093 eV/pixel		66 mrad		GIF Quantum ER	Energy scaled aligned using zero loss peak. Power law background removed.	
113	TbS-CrS2 Misfit Nanotube	S-L23, Cr-L23	TbS-CrS2	FEI Titan Low-Base	X-FEG	80 kV	0.6 eV	No	0.274 eV/pixel	STEM	31 mrad		Gatan Tridiem ESR 865	low loss + drift tube	
114	Niobium Oxide	O-K	Nb2O5	VG HB501UX	cold field emission	100 kV	1.3 eV	No	0.5 eV/pixel		6.5 mrad	5 secs	Parallel: Gatan ENFINA		0.54 t/λ
115	Niobium Oxide NbO2	O-K	NbO2	VG HB501UX	cold field emission	100 kV	1.3 eV	No	0.5 eV/pixel		6.5 mrad	5 secs	Parallel: Gatan ENFINA		0.58 t/λ
116	Titanium	Ti-L23	Ti	VG HB501UX	Cold FEG	100 kV		No	0.1 eV/pixel		6.5 mrad		Parallel: Gatan ENFINA		
117	Nickel Titanium	Ti-L23, Ni-L23	Ti3Ni4	CM30	feg Schottky	300 kV	1 eV		1 eV/pixel	Diffraction	4.01 mrad		Parallel: GIF200		0.376 t/λ
118	Titanium Dioxide (rutile Structure)	Ti-L23, O-K	TiO2	JEOL2010F	feg Schottky	200 kV	1.2 eV		0.2 eV/pixel	Diffraction	5 mrad	5 secs	Parallel: 766 Gatan		0.8 t/λ
119	Titanium Dioxide	Ti-L23	TiO2 (amorphous)	CM30	feg Schottky	300 kV	0.7 eV		0.05 eV/pixel	Diffraction	0.81 mrad	2 secs	Parallel: GIF200		0.492 t/λ
120	Titanium Dioxide	O-K	TiO2 (amorphous)	CM30	feg Schottky	300 kV	0.7 eV		0.05 eV/pixel	Diffraction	0.81 mrad	8 secs	Parallel: GIF200		0.492 t/λ
121	Titanium Dioxide	Ti-L23	TiO2 (anatase)	CM30	feg Schottky	300 kV	0.7 eV		0.05 eV/pixel	Diffraction	0.81 mrad	10 secs	Parallel: GIF200		0.257 t/λ
122	Titanium Dioxide	O-K	TiO2 (anatase)	CM30	feg Schottky	300 kV	0.7 eV		0.05 eV/pixel	Diffraction	0.81 mrad	20 secs	Parallel: GIF200		0.257 t/λ
123	Vanadium Oxide	V-L23	V2O5	Tecnai F20 monochrom	feg Schottky	200 kV	0.2 eV	Yes	0.02 eV/pixel		7.5 mrad		GIF: GIF Tridiem		
124	YAG (yttrium Aluminium Garnet)	O-K	Y3Al5O12	VG HB501UX	Cold FEG	100 kV	0.7 eV		0.1 eV/pixel	STEM	6.5 mrad	30 secs			
125	YAM (monoclinic Yttrium Aluminate)	O-K	Y4Al2O9	VG HB501UX	Cold FEG	100 kV	0.5 eV		0.1 eV/pixel	STEM	6.5 mrad	30 secs	Parallel: Gatan ENFINA		0.45 t/λ
126	YAP (yttrium Aluminium Perovskite)	O-K	YAlO3	VG HB501UX	Cold FEG	100 kV	0.7 eV		0.1 eV/pixel	STEM	6.5 mrad	40 secs	Parallel: Gatan ENFINA		
127	Zinc Oxide	O-K	ZnO	VG HB501UX	Cold FEG	100 kV	1 eV	No	0.671 eV/pixel		6.5 mrad	10 secs	Parallel: Gatan ENFINA		0.42 t/λ
128	Zinc Sulfide	S-L23	ZnS	VG HB501UX	Cold FEG	100 kV	1 eV	No	0.674 eV/pixel		6.5 mrad		Parallel: Gatan ENFINA		
129	H-BN CVD	B-K	BN	FEI Titan 60-300	xFEG	60 kV	0.06 eV	Yes	0.05 eV/pixel	STEM	8.33 mrad	35 secs	GIF QUANTUM	zero + dispersion	
130	Alpha-Bi2O3	O-K	Bi2O3	Jeol ARM-200cF	cold FEG	200 kV	0.1 eV	No	0.1 eV/pixel	STEM	35 mrad		Quantum GIF	Dual EELS	
131	Polybutylene Terephthalate	C-K	(C12H1204)n	JEOL JEM 2100F	FEG	120 kV	0.9 eV	No	0.05 eV/pixel		100 mrad	10 secs	Gatan Enfina	zero+dispersion	0.91 t/λ
132	Acrylonitrile Butadiene Styrene Copolymer (ABS)	C-K	(C8H8.C4H6.C3H3N)n	JEOL JEM 2100F	FEG	120 kV	0.9 eV	No	0.05 eV/pixel		100 mrad	10 secs	Gatan Enfina	zero+dispersion	0.76 t/λ
133	Styrene Acrylonitrile Copolymer (SAN)	C-K	(C8H8)n-(C3H3N)m	JEOL JEM 2100F	FEG	120 kV	0.9 eV	No	0.05 eV/pixel		100 mrad	10 secs	Gatan Enfina	zero+dispersion	0.57 t/λ
134	V thin film	V-L23	V	JEOL	LaB6	200 kV	1.2 eV		0.2 eV/pixel	Diffraction	100 mrad		Gatan Enfina	Voltage tube	3.08 t/λ
135	V thin film	O-K	V	JEOL	LaB6	200 kV	1.2 eV		0.2 eV/pixel	Diffraction	100 mrad		Gatan Enfina	Voltage tube	3.08 t/λ
136	TiO2 thin film	Ti-L23	TiO2	JEOL	LaB6	200 kV	1.2 eV		0.2 eV/pixel	Diffraction	100 mrad		Gatan Enfina	Voltage tube	3.17 t/λ
137	TiO2 thin film	O-K	TiO2	JEOL	LaB6	200 kV	1.2 eV		0.2 eV/pixel	Diffraction	100 mrad		Gatan Enfina	Voltage tube	3.17 t/λ
138	Argon gas at 750 mtorr	Ar-L23	Ar	JEOL	LaB6	120 kV	1.2 eV		0.3115 eV/pixel	Imaging	1.9 mrad		Gatan Enfina	Voltage tube	0.04 t/λ
139	B particle suspended on holey C	B-K	B	JEOL	LaB6	200 kV	1.2 eV		0.5 eV/pixel	Diffraction	100 mrad		Gatan Enfina	Voltage tube	2.75 t/λ
140	BP particle on holey C film	P-L23	BP	JEOL	LaB6	200 kV	1.2 eV		0.5 eV/pixel	Diffraction	100 mrad		Gatan Enfina	Voltage tube	4.30 t/λ
141	Amorphous C thin film	C-K	C	JEOL	LaB6	200 kV	1.2 eV		0.5 eV/pixel	Diffraction	100 mrad		Gatan Enfina	Voltage tube	1.53 t/λ
142	CaCO3 thin film	C-K	CaCO3	JEOL	LaB6	200 kV	1.2 eV		1 eV/pixel	Diffraction	100 mrad		Gatan Enfina	Voltage tube	3.08 t/λ
143	CaCO3 thin film	Ca-L23	CaCO3	JEOL	LaB6	200 kV	1.2 eV		1 eV/pixel	Diffraction	100 mrad		Gatan Enfina	Voltage tube	3.08 t/λ
144	CaCO3 thin film	O-K	CaCO3	JEOL	LaB6	200 kV	1.2 eV		1 eV/pixel	Diffraction	100 mrad		Gatan Enfina	Voltage tube	3.08 t/λ
145	CoO thin film	Co-L23	CoO	JEOL	LaB6	200 kV	1.2 eV		1 eV/pixel	Diffraction	100 mrad		Gatan Enfina	Voltage tube	2.53 t/λ
146	CoO thin film	O-K	CoO	JEOL	LaB6	200 kV	1.2 eV		1 eV/pixel	Diffraction	100 mrad		Gatan Enfina	Voltage tube	2.53 t/λ
147	Slightly oxidized Cr thin film	O-K	Cr	JEOL	LaB6	200 kV	1.2 eV		1.01 eV/pixel	Diffraction	100 mrad		Gatan Enfina	Voltage tube	3.43 t/λ
148	S particles on holey C film	S-L23	S	JEOL	LaB6	200 kV	1.2 eV		0.5 eV/pixel	Diffraction	100 mrad		Gatan Enfina	Voltage tube	3.31 t/λ
149	Slightly oxidized Cr thin film	Cr-L23	Cr	JEOL	LaB6	200 kV	1.2 eV		1.01 eV/pixel	Diffraction	100 mrad		Gatan Enfina	Voltage tube	3.43 t/λ
150	Slightly oxidized Cr thin film	C-K	Cr	JEOL	LaB6	200 kV	1.2 eV		1.01 eV/pixel	Diffraction	100 mrad		Gatan Enfina	Voltage tube	3.43 t/λ
151	SiO2 foil, ion milled	Si-L23	SiO2	JEOL	LaB6	120 kV	1.2 eV		0.5 eV/pixel	Diffraction	100 mrad		Gatan Enfina	Voltage tube	2.26 t/λ
152	SiO2 foil, ion milled	C-K	SiO2	JEOL	LaB6	120 kV	1.2 eV		0.5 eV/pixel	Diffraction	100 mrad		Gatan Enfina	Voltage tube	2.26 t/λ
153	Sc2O3 thin film	Sc-L23	Sc2O3	JEOL	LaB6	200 kV	1.2 eV		0.1 eV/pixel	Diffraction	2 mrad		Gatan Enfina	Voltage tube	
154	NaCl particle on holey C film	Cl-L23	NaCl	JEOL	LaB6	1 kV	1.2 eV		0.5 eV/pixel	Diffraction	7 mrad		Gatan Enfina	Voltage tube	2.56 t/λ
155	Air at 8 torr	O-K	N2	JEOL	LaB6	100 kV	1.2 eV		0.5181 eV/pixel	Imaging	4.1 mrad		Gatan Enfina	Voltage tube	
156	MnO2 thin film	O-K	MnO2	JEOL	LaB6	200 kV	1.2 eV		0.5 eV/pixel	Diffraction	100 mrad		Gatan Enfina	Voltage tube	3.52 t/λ
157	MnO2 thin film	Mn-L23	MnO2	JEOL	LaB6	200 kV	1.2 eV		0.5 eV/pixel	Diffraction	100 mrad		Gatan Enfina	Voltage tube	3.52 t/λ
158	LiF evaporated onto thin C film	F-K	LiF	JEOL	LaB6	200 kV	1.2 eV		1 eV/pixel	Diffraction	100 mrad		Gatan Enfina	Voltage tube	3.20 t/λ
159	Fe2O3 thin film	O-K	Fe2O3	JEOL	LaB6	200 kV	1.2 eV		0.5 eV/pixel	Diffraction	1.5 mrad		Gatan Enfina	Voltage tube	3.17 t/λ
160	EELS Acquire	Mn-L23, O-K, Ni-L23, Fe-L2	LiNiMnCoO2	FEI Talos	LaB6	200 kV	1.2 eV		0.25 eV/pixel	Diffraction		5 secs	Gatan Enfina	Voltage tube	
161	CuO thin film	O-K	CuO	JEOL	LaB6	1 kV	1.2 eV		2 eV/pixel	Diffraction	100 mrad		Gatan Enfina	Voltage tube	3.08 t/λ
162	CuO thin film	C-K	CuO	JEOL	LaB6	1 kV	1.2 eV		2 eV/pixel	Diffraction	100 mrad		Gatan Enfina	Voltage tube	3.08 t/λ
163	CuO thin film	Cu-L23	CuO	JEOL	LaB6	1 kV	1.2 eV		2 eV/pixel	Diffraction	100 mrad		Gatan Enfina	Voltage tube	3.08 t/λ
164	CsF particles on a thin C film	F-K	CsF	JEOL	LaB6	1 kV	1.2 eV		1 eV/pixel	Diffraction	0 mrad		Gatan Enfina	Voltage tube	4.78 t/λ
165	Cu thin film	C-K	Cu	JEOL	LaB6	200 kV	1.2 eV		2 eV/pixel	Diffraction	100 mrad		Gatan Enfina	Voltage tube	3.09 t/λ
166	Cu metal thin film	Cu-L23	Cu	JEOL	LaB6	200 kV	1.2 eV		2 eV/pixel	Diffraction	100 mrad		Gatan Enfina	Voltage tube	3.09 t/λ
167	MgO particles on holey C film	O-K	MgO	JEOL	LaB6	200 kV	1.2 eV		2 eV/pixel	Diffraction	100 mrad		Gatan Enfina	Voltage tube	
168	Air at 8 torr	N-K	N2	JEOL	LaB6	100 kV	1.2 eV		0.5181 eV/pixel	Imaging	4.1 mrad		Gatan Enfina	Voltage tube	
169	NiO thin film	Ni-L23	NiO	JEOL	LaB6	200 kV	1.2 eV		1 eV/pixel	Diffraction	100 mrad		Gatan Enfina	Voltage tube	3.66 t/λ
170	Air at 8 torr	O-K	O2	JEOL	LaB6	200 kV	1.2 eV		0.2088 eV/pixel	Imaging	100 mrad		Gatan Enfina	Voltage tube	
171	Air at 8 torr	N-K	O2	JEOL	LaB6	200 kV	1.2 eV		0.2088 eV/pixel	Imaging	100 mrad		Gatan Enfina	Voltage tube	
172	S particles on holey C film	C-K	S	JEOL	LaB6	200 kV	1.2 eV		0.5 eV/pixel	Diffraction	100 mrad		Gatan Enfina	Voltage tube	3.31 t/λ
173	Sc2O3 thin film	O-K	Sc2O3	JEOL	LaB6	200 kV	1.2 eV		0.1 eV/pixel	Diffraction	2 mrad		Gatan Enfina	Voltage tube	3.92 t/λ
174	Si single crystal, ion milled	C-K	Si	JEOL	LaB6	200 kV	1.2 eV		0.5 eV/pixel	Diffraction	100 mrad		Gatan Enfina	Voltage tube	3.92 t/λ
175	Si single crystal, ion milled	O-K	Si	JEOL	LaB6	200 kV	1.2 eV		0.5 eV/pixel	Diffraction	100 mrad		Gatan Enfina	Voltage tube	3.92 t/λ
176	Si single crystal, ion milled	Si-L23	Si	JEOL	LaB6	200 kV	1.2 eV		0.5 eV/pixel	Diffraction	100 mrad		Gatan Enfina	Voltage tube	3.92 t/λ
177	NiO thin film	O-K	NiO	JEOL	LaB6	200 kV	1.2 eV		1 eV/pixel	Diffraction	100 mrad		Gatan Enfina	Voltage tube	3.66 t/λ
